# Review of the Development of an Unbonded Flexible Riser: New Material, Types of Layers, and Cross-Sectional Mechanical Properties

**DOI:** 10.3390/ma17112560

**Published:** 2024-05-26

**Authors:** Qingsheng Liu, Zhongyuan Qu, Feng Chen, Xiaoya Liu, Gang Wang

**Affiliations:** School of Mechanical and Electric Engineering, Soochow University, Suzhou 215131, China; qsliu@suda.edu.cn (Q.L.);

**Keywords:** unbonded flexible riser, deep-sea development, mechanical properties, composite armor layer, configuration

## Abstract

Unbonded flexible risers consist of several helical and cylindrical layers, which can undergo large bending deformation and can be installed in different configurations to adapt to harsh marine environments; thus, they can be applied to transport oil and gas resources from ultra-deep waters (UDW). Due to their special geometric characteristics, they can ensure sufficient axial tensile stiffness while having small bending stiffness, which can undergo large deflection bending deformation. In recent years, the development of unbonded flexible risers has been moving in an intelligent, integrated direction. This paper presents a review of unbonded flexible risers. Firstly, the form and properties of each interlayer of an unbonded flexible riser are introduced, as well as the corresponding performance and configuration characteristics. In recent years, the development of unbonded flexible risers has been evolving, and the development of machine learning on unbonded flexible risers is discussed. Finally, with emphasis on exploring the design characteristics and working principles, three new types of unbonded flexible risers, an integrated production bundle, an unbonded flexible riser with an anti-H_2_S layer, and an unbonded flexible riser with a composite armor layer, are presented. The research results show that: (1) the analytical methods of cross-sectional properties of unbonded flexible risers are solved based on ideal assumptions, and the computational accuracy needs to be improved. (2) Numerical methods have evolved from equivalent simplified models to models that account for detailed geometric properties. (3) Compared with ordinary steel risers, the unbonded flexible riser is more suitable for deep-sea resource development, and the structure of each layer can be designed according to the requirements of the actual environment.

## 1. Introduction

**Figure 1:**
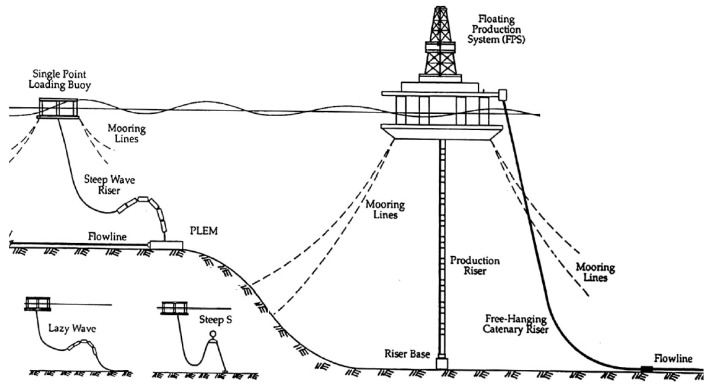
Sketch of riser system.

Riser systems (see Figure 1) are the key components of the offshore oil and gas extraction process, essentially referring to the conduit system that connects the surface floating unit to subsea equipment, which is required for the extraction of any offshore oil and gas resources. Offshore risers can be categorized in a variety of ways, and functionally, they can be simply divided into production riser systems and drilling risers. Among them, production risers have many structural forms, such as flexible risers, top tensioned risers (TTRs), steel catenary risers (SCRs), hybrid tower risers, and so on. With the advancement of science and technology, the operating water depth is increasing, and new riser structures come into being, such as compliant vertical access risers (CVARs), hybrid risers with multiple holes, etc. [1]. Since flexible risers have advantages, such as low bending stiffness, resistance to large deformation, corrosion resistance, ease of installation, and recyclability, they are prioritized or must be used for the extraction of offshore oil and gas resources in many cases [2,3,4,5]. In practical industrial applications, approximately 85% of dynamic risers are flexible risers [6].

**Figure 2:**
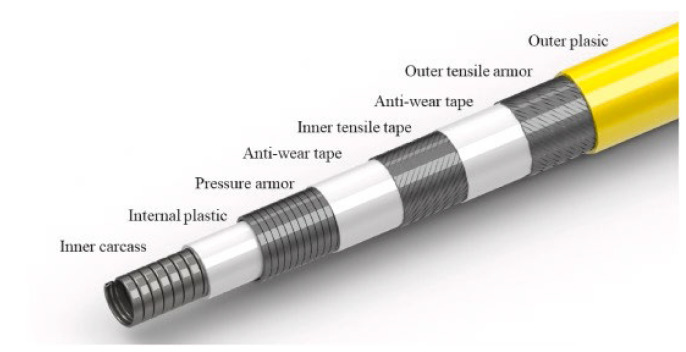
Sketch of unbonded flexible riser [11].

Flexible risers can be subdivided into bonded flexible risers and unbonded flexible risers (see Figure 2), both of which are multilayered composite structures containing an indefinite number of laying layers with different geometries, material properties, and functions (e.g., compressive armoring, tensile armoring, protective armoring, etc.) to ensure the safety of the riser during installation and operation. Flexible risers have flexible arrangement and configuration and can, therefore, be used in a wide range of applications, not only for the transmission of liquefied natural gas in close proximity (thermal insulation structure) but also as a long-distance oil pipeline. At the same time, they can be used as a transmission medium for cables (an integrated production bundle, IPB), which is a key piece of equipment in the field of offshore engineering. The difference between the two types of risers is that bonded flexible risers are laid by physical extrusion or chemical methods (e.g., vulcanization of rubber), in which interlayers cannot be separated or slip due to external forces. Bonded risers are commonly used as jumper hoses to transport resources between short distances [7,8]. An unbonded flexible riser, on the other hand, directly sets the layers in sequence. The following assembly may even generate interlayer gaps, and slipping between adjacent layers can occur under external forces. This type of riser is commonly used for long-distance oil and gas transportation [9,10]. The special structural form leads to the complex cross-sectional mechanical properties of unbonded flexible risers, especially when subjected to bending loads due to the relative slip of the internal spiral layers (mainly the tensile armored layers), which can lead to a sharp decrease in bending stiffness and the hysteresis phenomenon.

In recent years, the marine oil and gas industry has experienced a significant shift towards deep-water operations, which has led to increased demand for robust and sophisticated technology and equipment. The design of unbonded flexible risers has become particularly critical due to the challenges posed by deeper waters, such as higher pressures and the need for precise buoyancy control. A key factor in this design process is the suspended weight of the riser, which has prompted the exploration of novel materials to meet these stringent requirements.

One such innovation is the introduction of high-strength composite materials, including carbon fiber composites, which are now being utilized in the tensile armor layer of unbonded flexible risers. These materials not only ensure that unbonded flexible risers possess the necessary strength but also significantly reduce the weight of the tensile armor layer and the overall riser system. Additionally, composite cylindrical layer structures, such as Kevlar 49, are being employed to prevent radial failure, commonly referred to as birdcage failure, and to serve other functions like thermal insulation, which, in turn, influences the cross-sectional mechanical properties of unbonded flexible risers [12,13].

The integration of composites in unbonded flexible riser manufacturing is seen as a promising development within the industry, offering enhanced performance and reliability in deep-water environments. However, the material properties of these composites and the geometrical properties of unbonded flexible riser layers are complex and present a significant area of focus for future research and development. As the industry continues to push the boundaries of deep-water exploration, understanding and optimizing these properties will be pivotal in shaping future trends and advancements in marine oil and gas extraction technology.

## 2. Form, Performance Characteristics, and Configuration Characteristics of an Unbonded Flexible Riser

### 2.1. Form of Interlayers of an Unbonded Flexible Riser

**Figure 3:**
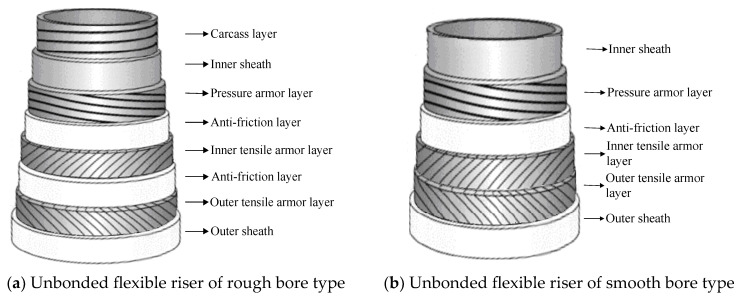
Different types of unbonded flexible risers [14].

**Figure 4:**
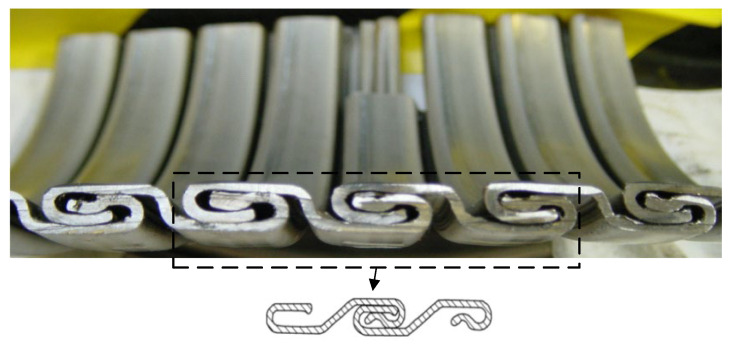
Sketch of the cross-section of the carcass layer.

**Figure 5:**
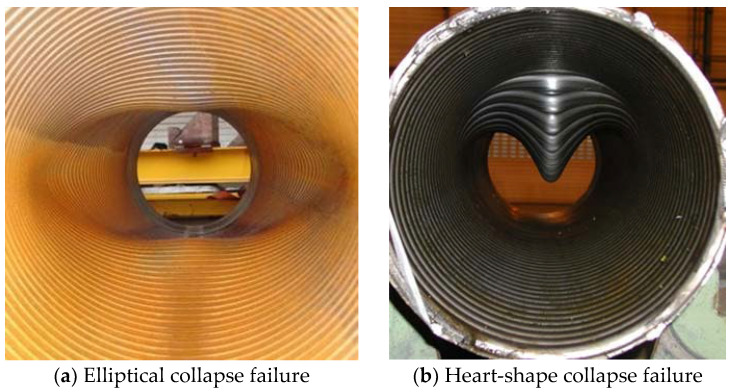
Collapse modes of the carcass layer [15].

**Figure 6:**
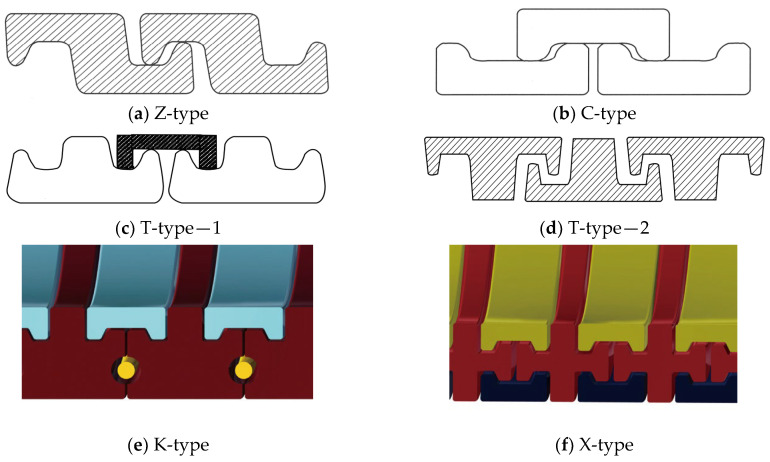
Profiles of the pressure armor layer.

**Figure 7:**
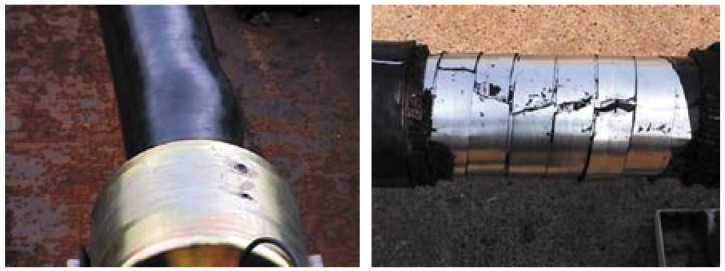
Burst of the pressure armor layer.

**Figure 8:**
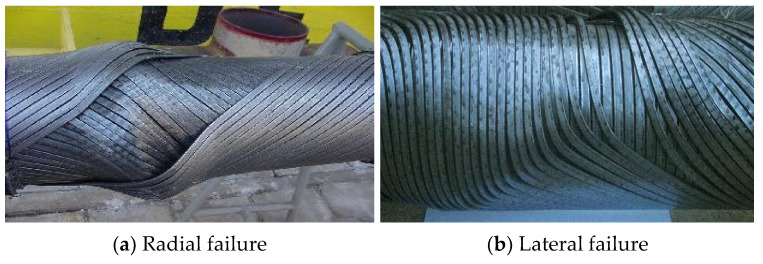
Burst of the pressure armor layer [22].

**Figure 9:**
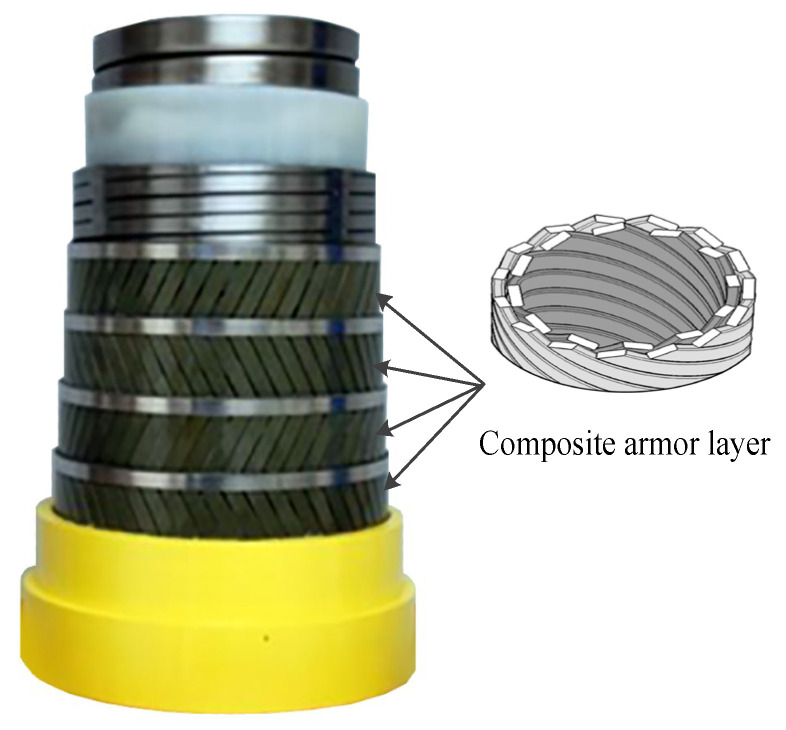
Unbonded flexible riser with a composite armor layer [24,25].

Unbonded flexible risers have been widely used in the offshore oil and gas industry since their inception in the 1970s. The internal layers of unbonded flexible risers can be assembled in a certain order; typically, the innermost layer is the carcass layer that bears the external compressive load and prevents damage during the manufacturing process. This type of unbonded flexible risers are also known as rough bore-type unbonded flexible risers (shown in Figure 3a), whereas unbonded flexible risers used in shallow waters do not generally contain this layer of structure, and are known as smooth bore-type unbonded flexible risers (as shown in Figure 3b). The interlayers of unbonded flexible risers can be divided into cylindrical and helical layers. Helical layers typically include a carcass layer, a pressure armor layer, and a tensile armor layer, and cylindrical layers include an internal and external sheath. Conventional unbonded flexible risers typically contain only metal layers and polymer layers. Based on the structure shown in Figure 3a, together with the development of an unbonded flexible riser, the geometric and material properties of each layer, as well as some functional layer and newly proposed layers, are presented from the inner to the outer layers:

1. Carcass layer (Figure 4). The carcass layer is typically composed of carbon steel, stainless steel (austenitic stainless steel AISl304, 304L, 316, 316L, or duplex stainless steel, etc.), corrosion-resistant alloys (resistant to the corrosive effects of H_2_S, CO_2_, CL ions, etc.), and other metal materials. The self-locking, non-watertight structure consists of steel material wound at a laying angle of close to 90°, and the cross-section is usually S-shaped, as shown in Figure 4. The special geometric form makes the axial tensile stiffness and torsional stiffness of the skeleton layer very small while ensuring sufficient bending performance but also preventing compression failure caused by external compressive loads. In general, as shown in Table 1, the skeleton layer of unbonded flexible risers features specific dimensional specifications tailored to risers of varying sizes. The typical failure modes of the carcass layer, as well as the pressure armor layer, are presented in Figure 5 [15].

2. Inner sheath. The internal sheath layer is a closed cylindrical shell structure extruded from polymers, usually nylon 11 (PA-11), nylon 12 (PA-12), cross-linked polyethylene (XLPE), high-density polyethylene (HDPE), etc. The thickness of this layer is usually 5.5 to 18 mm and is used for conveying medium (oil and gas resources). During the design process, the inner sheath layer is mainly considered with the compatibility of the fluid medium, fluid permeability, mechanical and thermal properties, and other factors. It should be noted that there may be gaps between the internal carcass layer and the inner sheath layer, which can lead to an infiltration phenomenon where gaseous substances accumulate in the annular region between these two layers [16].
3. Pressure armor layer [17,18,19,20,21]. The geometric properties of the pressure armor layer are similar to that of the carcass layer, which is a steel self-locking structure with a high laying angle, where the axial tensile and torsional stiffness can be basically ignored. The thickness of the layer is 4 to 12 mm, and the section geometry is mainly of the Z-type, C-type, or T-type (such as Figure 6a–d). The pressure armor layer mainly provides the radial stiffness of the unbonded flexible riser and carries the riser’s internal and external pressure (mainly carrying the internal pressure load caused by the internal medium). In cases where the riser is subjected to less internal pressure, the riser design can similarly eliminate that layer of the structure. The “Z-type” pressure armor layer is the main cross-section in the unbonded flexible riser industry. The specification parameters and scope of application of the pressure armor layer are presented in Table 2. In addition, there are some special cross-section shapes of the pressure armor layer, as shown in Figure 6e,f. These two structural forms of fatigue performance assessment are complex and do not promote application. Unlocking is the most common failure mode of tensile armor layers due to the fact that they are not completely self-locking like the skeleton layer structure and the asymmetry of the structural cross-section profile. The bearing ability of internal pressure is one of the most important properties of unbonded flexible risers. The burst failure of the pressure armor layer is illustrated in Figure 7.

4. Anti-friction layer: The anti-friction layer is made of polymer winding or directly formed by a non-metallic thermoplastic sheath cylindrical shell layer, usually PA11/6, PVDF, PP, etc. The anti-friction layer is located between the metal layers, mainly used to reduce and prevent friction between the metal layers, which can enhance the fatigue life of the pipeline and prolong its use.

5. Tensile armor layer. The tensile armor layer is the most important component layer of an unbonded flexible riser. A traditional unbonded flexible riser tensile armor layer consists of double or four layers of steel tendon with rectangular cross-sections, which are relatively wound. Additionally, the tensile armor layer is mainly used to provide axial stiffness, bearing the role of axial tension and torque, which can ensure the safe operation of the riser when it is subjected to self-weight and other external loads. In order to balance the role of tensile force and circumferential stress, the laying angle of the tensile armor layer can generally vary from 20° to 55°, and the gap between tendons generally accounts for 7~11% of the annular area of the tensile armor layer. The ability of the tensile armor layer directly determines the structural safety of the unbonded flexible riser. Additionally, structural failure is mainly caused by axial force, which mainly contains three failure modes: fracture failure, radial failure (birdcage failure, caused by axial compression, see Figure 8a), and lateral failure (caused by axial compression and wet environment, see Figure 8b).
6. Other intermediate layer structures (cylindrical layer). The intermediate layer of the unbonded flexible riser can be flexibly designed according to the needs of the actual situation. Additionally, according to the function of these cylindrical layers, they can be divided into an auxiliary layer, a bending-resistant layer, a thermal insulation layer, etc. Most of the intermediate layer structure is composed of a composite material cylindrical layer, such as a H_2_S gas corrosion-resistant layer and a thermal insulation layer. Some unbonded flexible risers add a layer of higher-strength composite material (typically Kevlar 49) as an anti-birdcage tape on the outside of the tensile armor layer to prevent radial failures from occurring [12].

7. Composite tensile armor layer [23,24,25,26,27,28]. The composite tensile armor layer is the application of composite materials to manufacture the tensile armor layer structure; its geometry is consistent with the traditional steel tensile armor layer. A schematic diagram containing a composite tensile armor layer of an unbonded flexible riser provided by Technip is presented in Figure 9. Offshore engineering towards ultra-deep waters is the reason that composite armor layers are generated. Beyond 2000 m of water depth, the weight of conventional flexible pipe becomes critical not only for the pipelay equipment and vessel but for the production floater as well. Substituting tensile steel armor with composite armor made from fiber-reinforced polymer has the potential to significantly reduce the weight of the pipe. Due to the high strength/mass ratio of carbon fiber, it can greatly reduce the overall quality of the unbonded flexible riser and improve the fatigue performance of the riser while meeting the strength; at the same time, compared with the steel structure, the composite material has better corrosion resistance, which can reduce the corrosive effect of chemicals and seawater on the risers in oil and gas fields, and it is the future direction of the development of unbonded flexible risers.

### 2.2. Performance Characteristics

**Figure 10:**
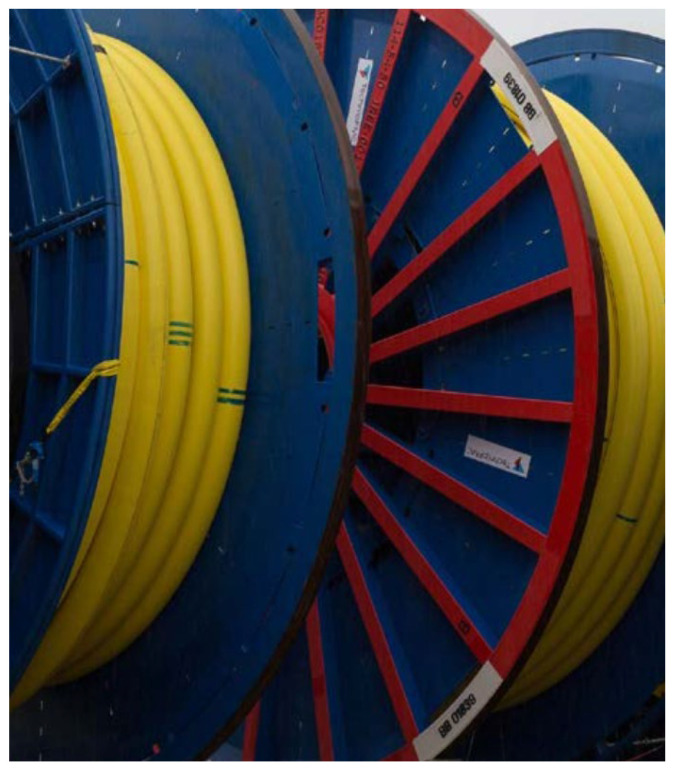
Sketch of a flexible pipe on the winch.

Compared to traditional steel risers, unbonded flexible risers containing multilayer composite structures have the advantages of corrosion resistance, fast installation, good flexibility for large deflection deformation, resistance to large internal and external pressures, high reliability, modular design, low maintenance cost, recyclability, etc. Depending on different needs, the number of interlayers of unbonded flexible risers can vary from the simplest of 4 layers to the most complex of 19 layers. Some performance characteristics can be drawn as follows:

1. Flexible. The interlayers of an unbonded flexible riser are relatively independent of each other, and the layers can slip relative to each other; thus, flexibility is the most prominent characteristic of the riser. For example, a typical unbonded flexible hose with an inner diameter of 203.2 mm has a safe bending radius of 2 m. It can fit the seabed terrain well without a large overhang and can be used in floating systems or on an uneven seabed. It is also easily wound, transported, and installed [13,29].
2. Convenient installation. Despite the high costs of unbonded flexible risers, installation costs are relatively low. Since conventional steel pipe laying costs are high, the sea welding inspection workload is large, and the laying cycle is long. An unbonded flexible riser has laying safety, convenience, and fast features, meaning the risers can be laid using an ordinary power positioning vessel, and the laying cost of construction compared to conventional steel pipe is reduced by more than 50%. The pipe is continuously wound on a winch, and the entire length of the pipe can be several thousand meters long (see Figure 10). The laying cycle is short, the offshore connection workload is small, and the laying speed is generally 500 m/h on average, which is more than five times the speed of steel pipe laying. Separate sections can be placed on the deck, and connections can be synchronized during installation without the need for other riser-based structures or underwater connection aids.

3. Modularization. The interlayers of unbonded flexible riser construction are independent of each other, making it possible to realize the exact requirements of a specific development. A simple riser for medium pressure resistance requires only 4 layers, while the most complex riser can have up to 19 layers. In addition to basic liquid leakage and anti-pressure layers, other layers can be sandwiched between the helical steel layers for abrasion resistance or thermal insulation. The modularity of the riser production process facilitates the adjustment of the thickness, cross-sectional shape, and number of layers to meet the different requirements of the customer.
4. Low corrosion-resistance requirement for steel helical tendons. Since the steel tensile armor of an unbonded flexible riser does not come into direct contact with the conveyed liquid, it does not need to have the same corrosion resistance as a conventional steel pipe. However, the innermost carcass layer of the hose must be in contact with the conveyed liquid and must be corrosion-resistant.
5. High pressure resistance. Unbonded flexible hose can have both internal and external pressure due to the specific configuration of the carcass layer, anti-pressure armor layer, and tensile armor layer.
6. Long production cycle life and low maintenance. Conventional steel pipe requires a large number of welded connections, while a sperate unbonded flexible riser is very long with fewer connecting joints in the whole riser, which is not needed for maintenance and repair of welds after being put into production and would not affect the production operation under adverse sea conditions. Additionally, the unbonded flexible riser would meet the requirements of continuous production, which has a longer fatigue life than that of steel pipe.
7. Recyclable. An unbonded flexible riser is easy to recycle and reuse, thus increasing the deep-sea oil field investment overall and is conductive to the environment.

### 2.3. Configuration Characteristics

**Figure 11:**
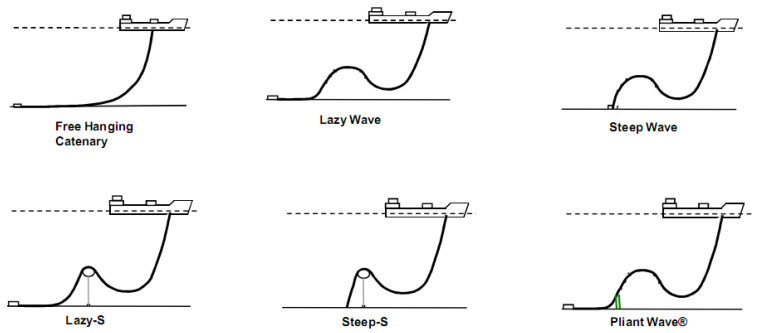
Configurations of an unbonded flexible riser.

Marine dynamic risers require careful design to prevent excessive bending. This design process involves line configuration, which takes into account the riser’s own gravity, buoyancy, and the constraint forces as presented by its overall shape. As depicted in Figure 11, there are various configurations, ranging from relatively simple to more complex. The simplest form is the free-hanging catenary configuration. This basic design is then adapted into more intricate shapes to meet specific design and operational needs. The configurations evolve from the catenary to include the following: the lazy wave configuration, which offers a more relaxed curvature; the steep wave configuration, characterized by sharper bends; the lazy-S configuration, which introduces an S-shaped pattern with a gradual transition; the steep-S configuration, featuring a more pronounced S-shape with abrupt changes in direction; the pliant wave configuration, which provides flexibility to adapt to various marine conditions. Each of these configuration designs is tailored to different requirements, ensuring that dynamic risers can function effectively in diverse marine environments [30].

**Figure 12:**
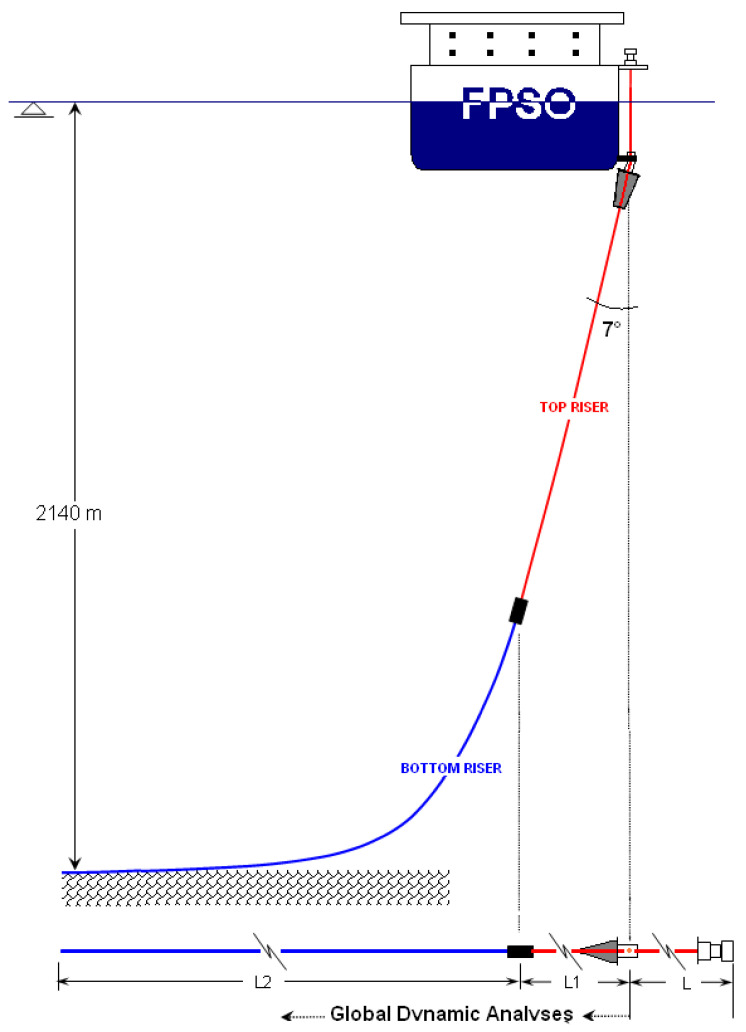
Sketch of a flexible pipe on the winch.

A typical configuration of an unbonded flexible riser with composite tensile armor layers is shown in Figure 12, wherein the top riser is the unbonded flexible riser containing the composite material tensile armor layer, and the bottom riser is the conventional unbonded flexible riser containing steel tensile armor layers. In deep water situations, unbonded flexible risers arranged in this way can significantly reduce the tensile forces on the top floating platform.

Risers and pipelines can be designed with reference to a variety of codes and standards from organizations such as classification societies and petroleum associations [31,32,33,34,35,36,37]. For unbonded flexible risers, the widely used codes are API 17B and API 17J, proposed by the American Petroleum Institute (API) [9,10], of which API 17B (Recommended Practice for Flexible Pipe) provides guidance on the design, analysis, fabrication, testing, installation, and operation of risers; API 17J (Specification for Unbonded Flexible Pipe) is the relevant standard specifically for unbonded flexible risers and includes guidance on safety, functionality, and dimensional definition. In addition, reference can also be made to standards such as DNV-OS-F201 (Dynamic Risers) from Det Norske Veritas [37].

## 3. Development of Cross-Sectional Properties of an Unbonded Flexible Riser

An unbonded flexible riser is a composite structure containing multiple layers, and there is also complex contact and friction between different layers. Under the action of external complex loads, the interlayers would be in contact or detachment with each other, and the tendons within the tensile armor layers would also undergo relative slippage, which makes solving the cross-section mechanical properties of unbonded flexible risers particularly complex. Additionally, the nonlinear cross-sectional properties of unbonded flexible risers have always been a hotspot and a difficulty in the research of the offshore oil and gas industry [6]. Structures such as umbilical cables and submarine cables also have similar structures to unbonded flexible risers, and even some unbonded flexible risers may contain a cable layer inside due to some special applications [38].

In terms of the research type, the mechanical properties of unbonded flexible risers can be mainly divided into the mechanical properties under axisymmetric load (axial tension, pressure, internal and external pressure, torque, and other loads alone or jointly) and the mechanical properties under bending load. When the material of the unbonded flexible riser changes only in the elastic range, the strain–load curve changes linearly when it is only subjected to axisymmetric loading [6,7]. Under a bending moment, the tendons within helical layers (mainly referring to the tensile armor layer) undergo relative slipping when the bending moment increases, which makes the bending stiffness of the unbonded flexible riser decrease sharply and then the hysteresis phenomenon occurs [39,40,41,42,43].

The mechanical properties of unbonded flexible risers are mainly studied by theoretical, numerical, and experimental methods. Although the test method can truly reflect the mechanical properties of unbonded flexible risers, the structure of unbonded flexible risers is relatively complex, the costs of unbonded flexible riser specimens are high and rare, and sometimes special test equipment is also needed to simulate external pressure or carry out the test. Thus, it is difficult to determine the detailed geometric and material properties of the specimen of unbonded flexible risers in published papers, and there are fewer corresponding data; so, at present, theoretical and numerical methods are the most important research methods used to study the mechanical properties of unbonded flexible risers.

Each of the three research methods has its own characteristics: the theoretical method is based on relatively strict assumptions and can be quickly calculated to obtain the approximate mechanical properties of unbonded flexible risers. In recent years, numerical methods, due to the advancement of computer technology, have been developed from equivalent simplified models to detailed geometrical models, and some new computational models have been proposed based on some assumptions for calculating the failure characteristics of unbonded flexible risers. The test methods also develop from initial indoor tests to the real sea dynamic test to verify the feasibility of full-size unbonded flexible risers [6,7,44]. The research progress on the cross-section mechanics of unbonded flexible risers is described in detail below.

### 3.1. Development of Unbonded Flexible Riser under Axisymmetric Loads

#### 3.1.1. Theoretical Method

The theoretical approach is generally based on some strict assumptions for the solution. Firstly, it is assumed that axisymmetric loads and the bending moment acting on the unbonded flexible riser can be decoupled [45,46], which is the basis of the solution. Secondly, the influence of the end boundary conditions is generally ignored in the theoretical model It is considered that the layers still comply with the assumption of flat cross-section under the action of the axisymmetric load [38] and show the same axial strains and torsion angles [47,48,49]. In contrast, factors such as initial defects of the layers of unbonded flexible risers [50,51] are neglected. Finally, the solution is based on the functional principle or the force balance differential equation [40,47,48,52,53].

Early theoretical models of unbonded flexible risers under axisymmetric loading were often specific to a particular mechanical property of the unbonded flexible riser. Féret and Bournazel [54] presented an analytical method to quickly assess the stress of the helical tendon, ignoring the effects of internal and external pressures and interlayer gaps in the unbonded flexible riser model. It was concluded that the non-metallic cylindrical shell layer only transmits interlayer pressures and ignores the role of its axial stiffness. Goto et al. [55] provided a theoretical model for evaluating the ultimate strength of unbonded flexible risers under axisymmetric loading and presented the corresponding simple formulae. Berge et al. [36] proposed a fast method for calculating the overall response of an unbonded flexible riser and presented expressions for the stress–load relationship under separate actions of axial force, internal pressure, and torque, respectively; however, the theoretical method is only applicable to separate load actions, and the response characteristics of each layer cannot be decoupled.

Subsequent studies have begun synthesizing theoretical models for unbonded flexible risers under multiple load conditions. McNamara and Harte [56] proposed a theoretical model that takes into account the effects of axial tension, torque, bending moment around the axis, and internal and external pressures, deriving stiffness, load, and displacement matrices for each layer based on the principle of fictitious work, and then assembling them to form an overall matrix, but ignoring variations in tube thickness. Later, Harte and McNamara [57] improved the accuracy of the analytical model by accounting for variations in tube thickness in their subsequent work. Witz and Tan [58] investigated the structural response of structures such as flexible risers, umbilical cables, and marine cables under axial and torsional loads based on the theory of nonlinear equations for classical spiral belts. Claydon et al. [59] divided the layers of unbonded flexible risers into helical layers and isotropic cylindrical shell layers and established a stiffness model including the relationship between the axial force–displacement and the internal pressure differential–radial displacement in the layers. Still, they ignored the effect of the interlayers’ contact within the unbonded flexible riser and considered that the axial force of unbonded flexible risers was the sum of the axial forces acting on each layer.

At present, the theoretical model of unbonded flexible risers under axisymmetric loading mainly considers the force and deformation relationship of each layer of the structure separately and, at the same time, considers the mutual contact effect between layers. Kebadze et al. [60,61] made a great contribution to the theoretical modeling of unbonded flexible risers under axisymmetric loading, and they summarized the theoretical models of their predecessors by dividing all the layers of unbonded flexible risers into cylindrical shell layers and helical layers, in which the cylindrical shell layer was analyzed using a thin-walled cylinder model, and the helical layer was divided into a helical layer with a regular cross-section (the tensile armored layer) and a helical layer with a non-regular cross-section. Assuming that each layer has the same axial elongation and torsion angle, considering the axial elongation and torsion angle of the overall unbonded flexible riser, as well as the thickness and radial strain of each layer, combined with the geometric relationship between the layers, the overall stiffness matrix is deduced through the functional principle, which can be used to arbitrarily solve the unknowns of each layer according to the force condition, determine the contact condition of neighboring layers, and calculate the effect of the interlayer contact pressure. Most of the subsequent research adopted Kebadze’s theoretical model. The subsequent work of Dong et al. [7,62] fully considered the tendon geometrical relationship of the helical layer accordingly. In addition, Bahtui [44], based on Kebadze’s theoretical model, simplified the helical layer structure with complex cross-sectional geometrical properties, such as the carcass layer and pressure armor layer, to an orthotropic anisotropic cylindrical shell. Ramos Jr. et al. [63,64] derived equilibrium equations for unbonded flexible risers under axisymmetric loading by a method similar to that of Kebadze, and presented in detail the assumptions of the theoretical approach: (1) the axial elongation and torsion angle are the same for all interlayers; (2) the interstitial gaps between the adjacent layers are not considered at the initial stage; (3) the inter contact between helical tendons in the tensile armor layer is not considered; (4) all materials are homogeneous, isotropic, and vary within the linear elastic range; (5) all strains are sufficiently small compared to the unit (small strain assumption, geometrically linear assumption); (6) initial defects arising from fabrication are ignored; (7) thickness varies uniformly in each layer; (8) for the helical tendons in the tensile armor layers, only the tangential and normal stresses to the direction of the interstitial axes are accounted for; (9) the frictional internal energy due to the relative displacement between the layers is neglected.

In addition, some other studies have provided equilibrium equations for unbonded flexible risers under axisymmetric loading through the simplified mathematical models. Among them, Custódio and Vaz [65] used the Lamé and Clebsch-Kirchhoff equations to describe the equilibrium equations for cylindrical shell and helical layers, taking into account the nonlinearity of the material and the relative contact between neighboring layers and provided an explanation for the occurrence of interlayer gaps through the contact between the layers. However, their theoretical model was not able to take into account the effects of the local bending and torsion of the helical layers. Sævik et al. [66] established the static equilibrium equations of helical belts from a microscopic point of view and presented a theoretical model of a helical belt layer under axisymmetric loading by means of the relationship between displacement and strain. Li et al. [67] analyzed the ultimate bearing capacity of unbonded flexible riser layers under axial force and radial pressure based on the theoretical model of Claydon et al. [59]. Knapp [68] derived the structural response of aluminum and steel helical tendons under axisymmetric loading based on the cable structure. Sævik and Bruaseth [69] used the umbilical cable as a research object and derived a theoretical model of the umbilical cable under axisymmetric loading through the principle of virtual work. Liu et al. [70,71] considered the viscoelasticity and structural damping of the nonmetallic layer in the unbonded flexible riser and established its theoretical model under axisymmetric loading. Liu et al. [24] extended the theoretical model of the steel tensile armor layer to the model of the composite tensile armor layer, which can be used for the structural response calculation of unbonded flexible risers containing both steel and composite tensile armored layers under the action of axisymmetric loading. The accuracy was verified through an eight-layer unbonded flexible riser model.

#### 3.1.2. Numerical Method

Although the theoretical method can be used to facilitate rapid calculations of the cross-sectional mechanical properties of unbonded flexible risers, it does not truly reflect the stress–strain level of the internal layer structure of unbonded flexible risers due to various assumptions. Meanwhile, it is expensive to carry out the tests of unbonded flexible risers, and some tests require specialized test equipment; therefore, the numerical method is an effective alternative method of research.

Numerical methods generally use either specialized finite element software (Bflex, version 3.10, https://www.sintef.no/en/software/bflex-for-flexible-risers/) to simulate the tendon of tensile armor layers and calculate their stress levels or general-purpose finite element software (ANSYS, version 24 R1, https://www.ansys.com/, ABAQUS, version 2024, Abaqus Finite Element Analysis|SIMULIA-Dassault Systèmes (3ds.com), and MARC, version 2024.1, https://hexagon.com/products/marc, etc.) to build a complete three-dimensional numerical model to simulate the mutual contact effects between layers. The former is based on the bending beam model [70] to simulate the helical tendon, which is computationally efficient, while the latter method meshes the whole numerical model and takes into account the effects of nonlinear contact between and within layers, which is computationally relatively inefficient. Among them, computational analysis based on general-purpose finite element software is commonly used as a numerical method, and the numerical analysis model has gradually evolved from an equivalent simplified model to a three-dimensional model, taking into account detailed cross-section geometry. The progress of the numerical method under axisymmetric loading will be introduced in the following section.

**Figure 13:**
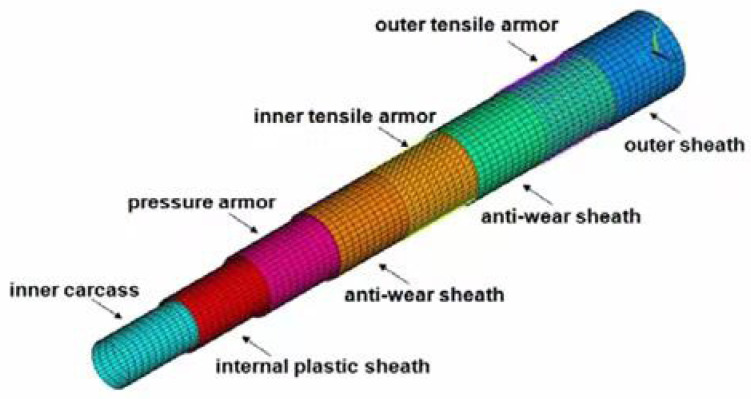
Simplified numerical model of an eight-layer unbonded flexible riser.

Numerical methods need to simulate the action of the layers of an unbonded flexible riser as much as possible, as well as the mutual contact between the layers; most of the early numerical models of unbonded flexible risers simplified the internal complex structure to some extent. Sousa et al. [72,73,74,75,76,77,78,79,80] carried out many studies on the mechanical properties of unbonded flexible risers based on numerical methods. They established a finite element model of unbonded flexible risers with a multilayered structure using ANSYS finite element software based on the study of Cruz [81], and investigated the structural responses under individual and combined axisymmetric loads, respectively. They divided the layers of the unbonded flexible riser into three types (Figure 13), in which the carcass layer and the pressure armor layer are simplified into an orthotropic anisotropic shell element, the nonmetallic cylindrical shell layer is simulated by an isotropic cylindrical shell element, and the tensile armor layer is simulated by an isotropic three-dimensional Euler beam element. This numerical model can be used to analyze the mechanical properties of the cross-section under axisymmetric loads, such as axial force and external pressure.

The tensile armor layer structure is the most important load-bearing structure in unbonded flexible risers, and different studies have proposed different treatments for this layer structure. Bahtui et al. [3,44,50,52,82,83] established the actual shape of the tensile armor layer based on ABAQUS software (version 2024) using an eight-node linear, reduced integrator element for simulation, with the multilayer modeling scheme taking into account the contact effect between the layers, which provides the possibility to analyze the hysteresis effect of unbonded flexible risers under bending moments. Vaz and Rizzo [29] simplified the tensile armor layer as an equivalent helical tendon, establishing a numerical model of a nine-layer unbonded flexible riser with high computational efficiency, which was modeled based using ABAQUS software (version 2024) with a lot of simplifications and assumptions. The spring element was used to simulate the supporting role of the internal and external structures of the two tensile armor layers, a cylindrical surface without thickness was established between two equivalent spiral steel strips to simulate the interlayer friction, and the friction coefficient was reduced to simulate the role of the anti-friction layer between the two layers. At the same time, a beam was established at the axisymmetric center of the unbonded flexible riser to simulate the axial and torsional stiffnesses of the skeleton layer, the internal sheath layer, the compressive armor layer, and the internal anti-friction layer. The authors also investigated the failure characteristics of the tensile armor layer under external pressure and axial compression based on the proposed numerical model.

The type of solution algorithm is crucial for solving the structural response of the numerical model of an unbonded flexible riser under external loading. Leroy and Perdrizet et al. [84,85] focused on explicit and implicit solution algorithms, and they developed a class of unbonded flexible riser numerical models containing five layers and equated the internal carcass layer, the inter sheath layer, and the pressure armor layer as a layer of cylindrical shell layer structure, the outer sheath structure of the tensile armor layer as a cylindrical layer, and the two tensile armor layers were simulated using body elements. Furthermore, the anti-friction layer structure between the two tensile armor layers was simulated using shell elements. The numerical model geometry is relatively simple and can be solved using both the explicit and standard algorithms of ABAQUS. In addition, Perdrizet et al. [84] conducted a more in-depth study on the algorithmic issues applied in the structural response calculations of unbonded flexible risers based on ABAQUS software (version 2024). The results showed that the use of the explicit algorithm needs to take into account the influence of inertial effects on the results of the calculations, and accordingly, the computation time is long. On the contrary, the standard algorithm has a higher solving efficiency, while the calculation is not easy to converge considering the geometric and material nonlinearities as well as the nonlinearities of interlayer and intralayer mutual contact. Thus, Perdrizet et al. [84] recommended using the explicit solving algorithm while taking into account computational time and computational efficiency. In recent years, some researchers have used the numerical method of taking into account the geometric properties of the detailed cross-sectional properties of unbonded flexible risers in multilayer composite structures, and due to the existence of complex contacts and strong geometric nonlinearities in the numerical model, the explicit algorithm is applied for the solution.

**Figure 14:**
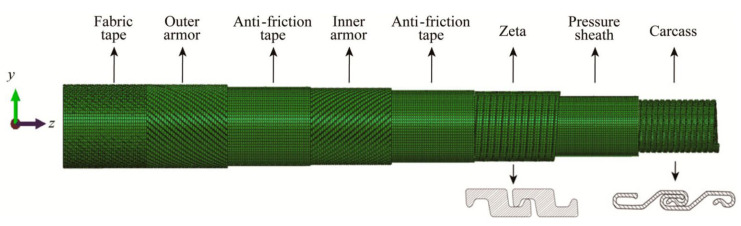
Numerical model of an eight-layer unbonded flexible riser with detailed carcass layer and pressure armor layer.

With the improvement of computer computational performance, more and more researchers began to consider the establishment of numerical models containing the detailed geometric characteristics of unbonded flexible risers. Among them, Li et al. [86,87] established a ten-layer unbonded flexible riser numerical model based on ABAQUS software (version 2024) containing detailed the geometric properties of the carcass layer and pressure armor layer, in which the carcass layer was simulated using a four-node reduced integral shell element. All other layers were simulated using an 8-node linear reduced integral body element. The structural response of this numerical model of unbonded flexible risers under axial tension and internal and external pressures was also investigated to evaluate the stress levels in each layer. Ren et al. [88] established a numerical model of a class of unbonded flexible risers with a 2.5-inch eight-layer structure based on ABAQUS software (version 2024), including the establishment of the S-type skeleton layer and the Z-type pressure-resistant armored layer containing all the geometric characteristics (e.g., Figure 4 and Figure 6). All the layer structures were simulated with the eight-node linearly reduced integration of the body element, and the amplification of the computation time was adopted to solve the problem of long calculation time. The step method is used to solve the problem of long calculation time, and the structural response of the unbonded flexible riser under axisymmetric load is verified by theoretical and experimental methods. The numerical model can effectively simulate the mutual contact and friction effects between and within the layers; therefore, it can be used to calculate the mechanical properties of the cross-section under axisymmetric and bending loads. Ren et al. [88,89,90] established a numerical model of a class of unbonded flexible risers with a 2.5-inch eight-layer structure based on ABAQUS software (version 2024), including the establishment of the S-type carcass layer and the Z-type pressure armor layer containing all the geometric characteristics (see Figure 14). All the layers were simulated linearly by the eight-node reduced integration of the body element. Meanwhile, the method of enlarging the calculation time step was adopted to solve the problem of long calculation time, and the structural response of unbonded flexible risers under axisymmetric loading was verified by theoretical and experimental methods. The numerical model can effectively simulate the mutual contact and friction effects between and within layers; therefore, it can be used to calculate the mechanical properties of the cross-section under axisymmetric and bending moments. Yoo et al. [91,92] established a carcass layer with detailed geometrical characteristics using ANSYS software (version 24 R1) and simplified it to an equivalent cylindrical shell layer by analyzing the mechanical failure characteristics of the carcass layer under axial force. Taking a class of eight-layer 2.5-inch unbonded flexible risers as an example, an equivalent simplified eight-layer numerical model and a five-layer numerical model (the internal four-layer structure was equivalent to one layer) were established, and the effect of simulating the cylindrical shell layer using a shell element or a body element was taken into account to study the axial load carrying capacity of the unbonded flexible risers. Zhang et al. [11] also developed a numerical model containing detailed geometries, and all the layer structures were also simulated using body elements and analyzed the structural response under external loads such as axial tension, torque, and internal and external pressures.

In summary, the establishment of a three-dimensional numerical model of an unbonded flexible riser under axisymmetric loading has roughly gone through development from simplifying part of the layer structure to taking into account the detailed geometrical properties of each layer, and due to the existence of a large number of geometrical nonlinearities and contact nonlinearities within the numerical model, it is generally solved by explicit algorithms.

#### 3.1.3. Test Method

There is a relative lack of research on the cross-section mechanical properties of unbonded flexible risers based on the experimental research method, partly due to the high cost of unbonded flexible risers and partly because some special test equipment is needed to carry out the tests. The load–strain relationship is basically linear, and the related experimental studies are relatively few compared to the mechanical properties under bending load, which is a strong nonlinear phenomenon.

**Figure 15:**
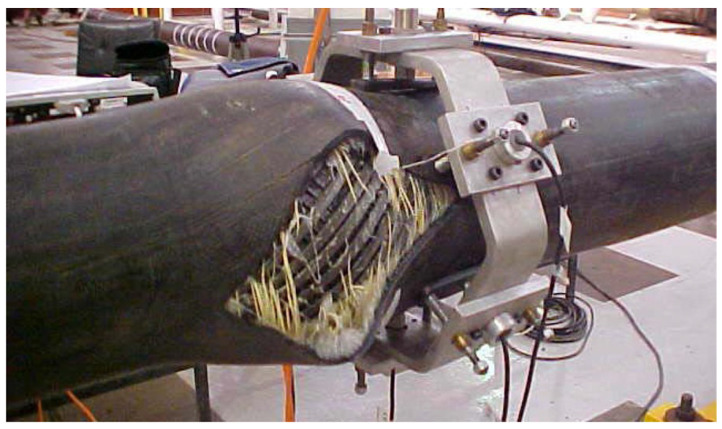
Radial failure of a seven-layer, 4-inch unbonded flexible riser.

Most of the model tests under axisymmetric loading are based on modeling a sufficiently long section of the unbonded flexible riser. Among them, Witz [14] carried out a class of eight-layer 2.5-inch unbonded flexible riser tests, which is the most representative test in the published literature so far, and he not only provided the geometrical and material properties of each layer of the unbonded flexible riser, but also elaborated the process of carrying out the test and the imposed boundary conditions. Furthermore, the test results were compared to the predictions provided by several research organizations; therefore, most of the subsequent theoretical and numerical method studies are based on this test as reference and validation, and the test results show that the experimental data of the structural response under axial tension and torque loading are in good agreement with the theoretical predictions. Ramos Jr. et al. [11,93] investigated the relationship between axial force and axial elongation of unbonded flexible risers by loading cyclic axial force, and the results showed that unbonded flexible risers also suffer from some nonlinear hysteresis under cyclic axial force. Vargas-Londoño et al. [94] found a similar phenomenon in a subsequent experimental study and found that the different spiral steel strips in the tensile armored layer did not have the same load-carrying capacity. Zhou et al. [95] investigated the effect of anti-friction layers in unbonded flexible risers based on experimental methods and also obtained a nonlinear relationship of load–strain under axisymmetric loading. Sousa et al. [96] investigated the structural response of a class of 4-inch, seven-layer unbonded flexible risers under axial pressure by experimental methods. The test results showed that the axial force–strain curve of the unbonded flexible riser varied approximately linearly before radial failure occurred, followed by the destruction of the outer sheath layer (shown in Figure 15) and radial failure of the riser.

In addition, Yue et al. [97] verified the mechanical properties of unbonded flexible risers in shallow water under axial tension through full-scale modeling tests. Sævik et al. [98] and Vaz et al. [99] carried out studies based on experimental methods for umbilical cable structures with similar properties to unbonded flexible risers and also found that the structures have certain axisymmetric loading with nonlinear properties.

### 3.2. Development of an Unbonded Flexible Riser under a Bending Moment

Under the action of a bending moment, the increasing curvature of the helical layer would gradually overcome the interlayer friction, and then relative slip would occur. Thus, the bending stiffness of the unbonded flexible riser decreases sharply, and the phenomenon of nonlinear hysteresis occurs, which has always been the hotspot and difficult point of academic research.

#### 3.2.1. Theoretical Method

The understanding of the slippage of spiral layer structures in unbonded flexible risers has gone through a process from simple to complex. Among them, Lanteigne [100] proposed the flat cross-section assumption in the bending model and argued that the bending stiffness of the helical layers is not fixed but depends on the radial forces and tensions in each layer by gradually overcoming the effect of friction and going through a transitional phase until the layers were free to slide between each other. At the same time, the tendons in different layers are subjected to different forces, and the slip starts from the outermost layer of the helical layer with different critical curvatures. Féret and Bournazel [54] derived formulas for the displacement and offset angle of a spiral belt along its own axial and lateral directions based on spatial geometric relationships, and they pointed out that the frictional moment between layers needs to be overcome before bending an unbonded flexible riser, providing a moment–curvature hysteresis curve. Mciver [101] introduced a theoretical modeling method for the structure of each layer separately, where the homogeneous layer is considered as a thick-walled continuum, and the helical layer is considered a bending beam with axial, bending, and torsional stiffness. The bending hysteresis characteristics of the riser were analyzed by considering the loads, including tension, torque, shear, and bending moment, as well as the effects of wall pressures, temperature differences, and friction. Ramos Jr. et al. [102] introduced the process of solving the bending stiffness of unbonded flexible risers, i.e., first assuming that the riser was in an unstressed state, after which axisymmetric loads (axial force, internal and external pressures, and torsion) were applied, and finally, bending moments are applied. As a result, the structural response characteristics of the un-slip and full-slip phases were solved. They ignored the bending stiffness of the partially slipped phase, which can be used to quickly calculate the bending stiffness in the full slip phase.

**Figure 16:**
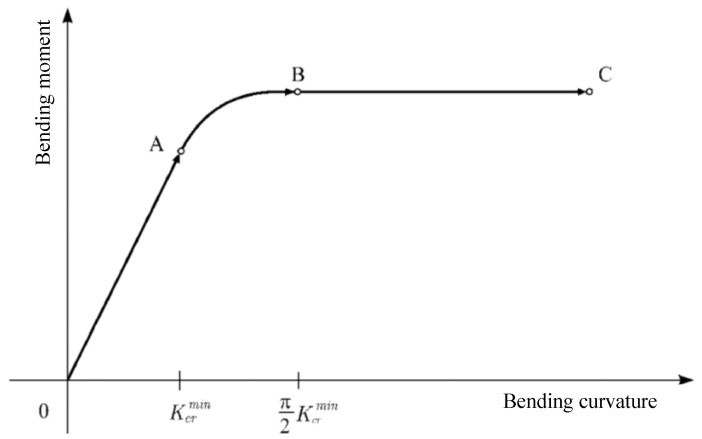
Hysteresis curve of the bending moment versus bending curvature.

**Figure 17:**
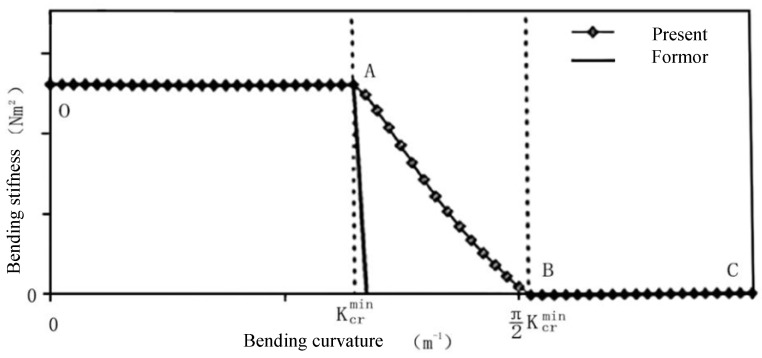
Bending stiffness versus bending curvature curve.

Subsequent studies assumed that the helical strip varies only along its own axis under the bending moment to give a slip model of the helical tendon, and the nonlinear moment–curvature relationship for some of the phases was given. Among them, Kebadze et al. [60,61] considered that the tensile armor layer, after overcoming the friction between the adjacent layers, transitions from the beginning of partial slip to full slip and summarized the bending process into three stages: no slip, partial slip, and full slip (see Figure 16). They ignored the mutual contact between the helical steel tendon of the tensile armor layer and provided an expression for the strain of the helical layer by means of a spatial geometric relation. Then, the expression between the bending moment–curvature was given by the functional principle in three stages, and the expression of the bending stiffness in each stage was presented, as in Figure 17. Firstly, the model under axisymmetric loading was used to solve the interlayer contact pressure, and the static friction was calculated by assuming that the pressure is constant. Then, the critical curvature was given as the calculation method: in the OA segment shown in Figure 16 $K\leq K_{cr}^{\min}$, the tensile armor layers have not been able to overcome the static friction between the layers, and there is no change in bending stiffness (segment OA of Figure 17). In the AB segment shown in Figure 16 $K_{cr}^{\min}<K<\pi \cdot K_{cr}^{\min}/2$, the unit at the neutral axis of the tendon reaches the critical curvature and starts to slip, while the other parts of the tendon are still subject to static friction, and gradually transition to the unit farthest from the neutral axis reaching the critical curvature, where the bending stiffness decreases nonlinearly (segment AB of Figure 17). In the BC segment shown in Figure 16 $K\geq \pi \cdot K_{cr}^{\min}/2$, the whole helical tendon has overcome the role of static friction. At this time, the strain of the tendon is completely determined by the static friction and, in the absence of consideration of the local bending and torsion of the spiral belt, the tensile armor layer completely loses bending stiffness, as seen in the BC section shown in Figure 17, and the corresponding bending stiffness is zero. Kebadze and Kraincanic [61] also provided formulas for calculating the bending stiffness induced by the effects of local bending and torsion of the helical tendon. Then, Dong et al. [7,62,103], on the basis of the theoretical model of Kebadze, accurately calculated the curvature and deflection changes of the helical tendon and regained the bending stiffness induced by the effects of local bending and torsion of the helical tendon. At the same time, they provided the explicit expression of bending stiffness in the partially slipping phase, which greatly simplified the calculation process. In addition, Wang et al. [104,105] analyzed the variation of stresses in helical belts under the action of irregularly varying bending moments and curvatures based on the theoretical models of Kebadze et al. [61] and Dong et al. [103].

In addition, some other research has developed equivalent mathematical and analytical models based on the nonlinear bending characteristics of unbonded flexible risers. Østergaard et al. [106] provided a mathematical expression for the free sliding of a helical belt in a frictionless cylindrical shell layer. Approximate expressions for the bending stiffness have also been presented by some riser manufacturers, e.g., Péronne et al. [107] and Zhang et al. [108]. Meanwhile, Witz and Tan [109,110] investigated the bending characteristics of marine and umbilical cable structures and proposed the theory that friction affects bending characteristics. They pointed out that slip is caused by an inhomogeneous axial deformation of the helices prior to the occurrence of the slip and that homogeneous axial deformation is expected along the deflected helices after the slip occurs. The effect of boundary conditions was also investigated, and it was proposed that when the ends are not subjected to any physical constraints, the helix would experience minimum uniform axial strain, and the helix at the ends would no longer be retained in the planar cross-section.

Bending hysteresis characteristics remain a hot topic for unbonded flexible risers [111,112,113,114,115,116]. For the tensile armor layer structure, current research mainly focuses on how to accurately describe the slip characteristics of the helical tendon using the following models: the tensile armor layer bending hysteresis model established by considering different friction models and based on the theory of bending beams [117,118]; the analytical analysis model considering the deformation characteristics of the tensile armor layer under the action of riser torque and bending around the axis [62]; the bending hysteresis model established by considering the effect of shear deformation of the cylindrical shell layer structure in the riser [119].

#### 3.2.2. Numerical Method

The numerical method used under the bending moment is analogous to the one used under axisymmetric load, which is also based on special finite element software or based on general-purpose finite element software using a layered modeling method for analysis. Additionally, the importance of interlayer contact and friction is mentioned in the above theoretical approach, which must be focused on in the numerical simulation.

**Figure 18:**
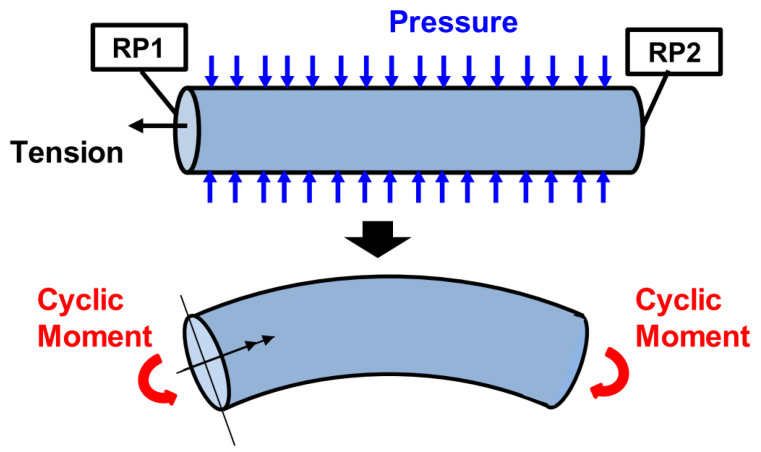
Loading sequence for combined loads [119].

Numerical methods for the bending characteristics of unbonded flexible risers have also evolved from equivalent simplified models to models that account for detailed geometric properties. Based on finite element software, different studies have established multi-layer numerical models of unbonded flexible risers to study their nonlinear bending characteristics. Most of them have simplified the numerical model [82,83]. Yun et al. [119] simplified the structure of the carcass layer and pressure armor layer and studied the bending characteristics of unbonded flexible risers by applying the axisymmetric load and then the bending moment (Figure 18). They investigated the effect of different axisymmetric loads on the bending characteristics, and the results of the calculations showed obvious hysteresis. The authors suggested that the bending characteristics of risers need to be taken into account segmentally when analyzing the dynamic response of unbonded flexible risers [120].

**Figure 19:**
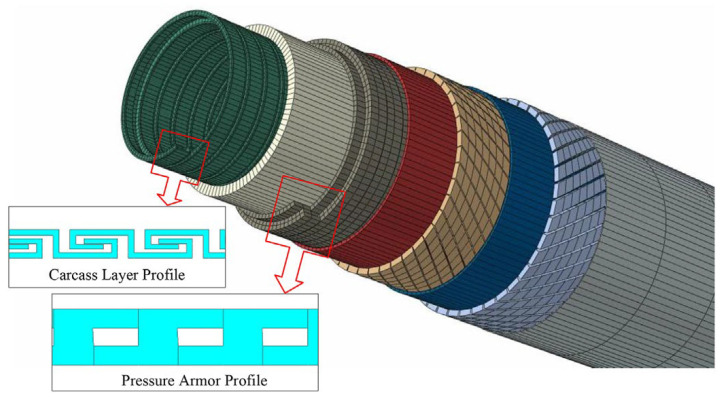
Finite-element model of Zhang et al. for an unbonded flexible riser [121].

The multi-layer modeling method considering detailed geometry can effectively simulate the complex contact between and within the helical layer (mainly the carcass layer and pressure armor layer); in contrast, the corresponding numerical method is difficult to model with more elements, the calculation time is too long, and it is easy to cause the results to be non-convergent. Few studies have carried out research on the cross-sectional mechanical properties of unbonded flexible risers under bending loads using this method. Among them, Zhang et al. [121] took a class of eight-layer 2.5-inch unbonded flexible risers as the object of study, and simplified the cross-sectional mechanical structure of the carcass layer and the pressure armor layer to some extent (shown in Figure 19). The mesh of the regular cylindrical shell layers was divided into large elements. The initial prestressing effect between the adjacent layers was given by applying a large external pressure (1.8 MPa) to the outermost sheath layer, after which the applied bending moment load showed significant hysteresis characteristics.

The development of numerical models of unbonded flexible risers containing detailed geometric properties to simulate the nonlinear hysteresis characteristics of the structure is the current direction of development [122,123,124,125]. The advantages of this include simulating the nonlinear contact and friction characteristics between adjacent layers and calculating the structural response characteristics of the unbonded flexible risers under the action of complex coupling loads. The disadvantages include strong geometric nonlinearity, the fact that numerical process calculations are not easy to converge, and the nonlinear contact within and between the layers in the model may lead to unacceptable computational time.

#### 3.2.3. Test Method

The structural response of unbonded flexible risers under the bending moment is complex, and the corresponding experimental studies are relatively few. Most of the open literature is characterized by some experimental data given by the riser manufacturers, and the detailed geometrical parameters and material properties of the risers are generally not given.

Among the tests, the most classical test of a class of eight-layer 2.5-inch unbonded flexible risers was carried out by Witz [14], who provided the structural response of unbonded flexible risers under cyclic bending loads and observed significant hysteresis characteristics. Meanwhile, Witz [14] also investigated the effect of internal pressure on bending, providing moment–curvature curves at an internal pressure of 30 MPa. They found that the bending stiffness of the complete slip phase decreased after the application of internal pressure, but this result is highly controversial. Magluta et al. [126], in a subsequent experimental study, found that bending stiffness was not affected by internal pressure, while Bech and Skallerud [127] and Troina et al. [128] also found that bending stiffness increased after the application of an internal pressure load by means of experimental methods. No recognized solution has yet been formulated to address this problem. With the exception of Witz [14], most of tests were conducted by riser manufacturers. For example, Zhang et al. [108] carried out an experimental study based on three different sizes of 4-inch, 10-inch, and 15-inch pipes, simplified the bending hysteresis model to a bifold model, and developed an approximate calculation method of equivalent non-slip stiffness and full slip stiffness considering parameters such as internal and external pressure, axial force, and interlayer friction coefficient. However, some parameters in this calculation method are obtained by experiments, and the parameter data are not given in the paper.

Overall, there are relatively few experimental studies on the bending hysteresis characteristics of unbonded flexible risers, but the test results all show the obvious hysteresis characteristics of unbonded flexible risers under bending loads.

### 3.3. Development of the Study of Typical Failure Characteristics of Unbonded Flexible Risers

Unbonded flexible risers transport marine oil and gas, and once they fail, they not only bring economic losses but they might even threaten the marine environment and cause serious consequences. The main failure modes of unbonded flexible risers are as follows: collapse failure of the carcass layer and pressure armor layer; burst failure of the pressure armor layer; fracture, lateral, and birdcage (radial) failure of the tensile armor layer, etc. The following is a brief introduction to these failure modes from the perspectives of the theoretical method, numerical method, and test method.

#### 3.3.1. Collapse Failure of the Carcass Layer and Pressure Armor Layer

The structure of the carcass layer is similar to that of the pressure armor layer, and the collapse failure mode is also relatively similar, as shown in Figure 5. The reasons for failure mainly include excessive axial tension or external pressure, excessive load during installation, breakage of the outer sheath layer, and initial defects in manufacturing [129].

Uniform external pressure or radial compression might lead to collapse failure. For uniform external pressure, the theoretical approach is mainly used to equate the carcass layer and the pressure armor layer as a circular structure with a rectangular cross-section, after which the differential equations of the deflection curves of the circular ring are established by solving the elastic stability theory to calculate the critical compression failure value [130]. Numerical methods are used to investigate the structural properties under uniform external or radial compressive loads by building numerical models taking into account the detailed geometry. Due to the symmetry of the structure, 1/4 or 1/2 circular numerical models or full-size models can be used for the solution [131,132,133,134,135].

#### 3.3.2. Burst Failure of the Pressure Armor Layer

Burst failure (see Figure 7) of compressive armor layers is caused by excessive internal pressure, which might also cause the failure of tensile armor layers.

There are relatively few studies on the burst failure of unbonded flexible risers. Theoretical models are mostly based on single-layer pipes, which are categorized into elastic failure, plastic failure, and ultimate failure according to the distribution state of cross-section stress and solved by yield conditions such as Mises’ criterion and Tresca’s criterion [1]. In addition, Yuan [136] investigated the limit state of unbonded flexible risers under internal compressive loading by a numerical method of multilayer modeling.

#### 3.3.3. Failure of the Tensile Armor Layer

The tensile armor layer bears the main axial force and torque of unbonded flexible risers, which is the most important layer in risers, and the failure modes can be classified into fracture failure, birdcage failure (radial failure), and lateral failure.

The tensile armor layer of unbonded flexible risers may suffer fracture failure when subjected to excessive tensile forces. The principle of failure is relatively simple, i.e., failure occurs when the tensile armor layer reaches its axial load-carrying capacity. Theoretical solution methods are based on the theoretical model of an unbonded flexible riser under axisymmetric loading, considering the elasticity and plasticity of the material of the tensile armor layer and calculating the stress, which is considered to lose its axial load carrying capacity by reaching plasticity [91]. Numerical methods often rely on simplified models or approximations to make complex calculations more tractable. Yoo et al. [91] investigated the ultimate carrying capacity under axial tension based on a class of eight-layer 2.5-inch unbonded flexible risers and simplified it to equivalent eight- or five-layer structures.

The failure modes of the tensile armor layer under axial compression are complicated since the special structural form of the unbonded flexible riser leads to its axial compressive stiffness being much smaller than the axial tensile stiffness, so it might fail when subjected to compression during the installation or life cycle. There are two failure modes under axial compression: radial failure and lateral failure, as shown in Figure 8 [24,137]. The cause of both failure modes starts from damage to the outer sheath layer. After damage to the outer jacket layer occurs, radial failure occurs in the tensile armor layer, which loses the radial constraints [138], and lateral failure might occur when there is sufficient radial support or when the friction between the layers is very small [139]. Lateral failure is more difficult to observe and is more hazardous compared to radial failure modes.

For theoretical methods, Sævik and Thorsen [140] presented a quick calculation of radial and lateral failure of tensile armor layers based on the bending beam theory and compared it to the test results, which showed that the theoretical predictions were relatively conservative. Østergaard et al. [141,142] provided a method for calculating lateral failure, and the theoretical results are also lower than those obtained through tests.

Numerical methods facilitated some simplifications of unbonded flexible risers, generally by simplifying the internal structure (carcass layer, pressure armor layer, internal sheath layer, etc.) into a rigid core layer [143,144,145], and the radial and lateral failure characteristics were investigated, taking into account the effects of material nonlinearity and other factors.

**Figure 20:**
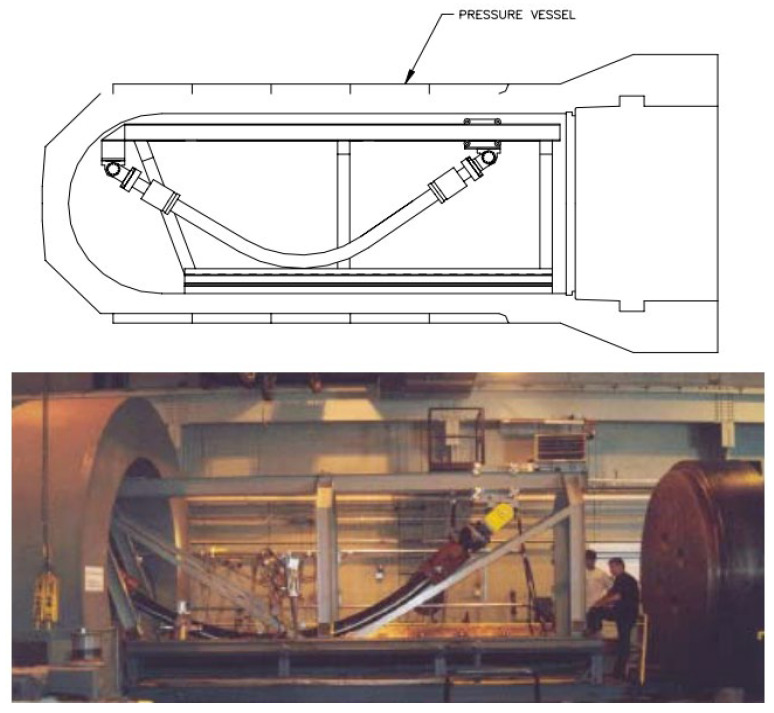
Profile of the pressure tank test [146,147].

**Figure 21:**
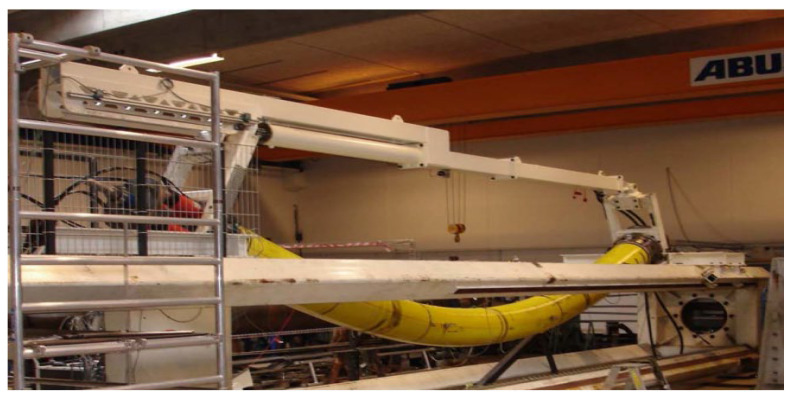
Compression test arrangement for an unbonded flexible riser [22].

The test methods for tensile armor layers under the axial compression can be divided into three forms: deepwater immersion tests (DIP), pressure tank tests (as shown in Figure 20 [146,147]), and mechanical performance experiments (as shown in Figure 21 [22]).

The DIP test uses a real-size unbonded flexible riser to carry out the test and requires the cooperation of specialized equipment such as underwater robots and pipe-laying vessels. Although detailed riser failure characteristics can be observed, the DIP test is extremely costly, has a long cycle time, and only very few riser manufacturers have the strength to carry out the DIP test at present. 

Pressure tank tests can simulate the wet environment of the ocean, and the cost is only 1/5 to 1/10 of that of carrying out DIP tests. Secher et al. [145] simulated the lateral failure characteristics of three different sizes of unbonded flexible risers, 7-inch, 9-inch, and 11-inch, respectively, by DIP and pressure tank tests. The results obtained from the two different test methods were found to be basically the same, and it was found that the larger the size of the unbonded flexible riser, the more prone it was to lateral failure.

Mechanical tests require relatively low levels of test equipment and can use X-rays to detect the occurrence of failure in the internal structure of unbonded flexible risers.

There is a rising trend of research on the failure of the tensile armor layer of unbonded flexible risers, and many researchers have also investigated the failure characteristics of the structure through different methods in recent years [148,149,150,151,152].

### 3.4. Development of Machine Learning Methods on Unbonded Flexible Risers

With the rapid development of information technology, the field of artificial intelligence has achieved impressive results, and the cross-study between machine learning methods (ML) and the traditional engineering industry has opened up a new way for solving complex engineering problems. Furthermore, a variety of data-driven algorithms, such as neural networks and support vector machines, etc., have been proven to have strong practical applications in marine and offshore engineering [153]. In the field of marine risers, the models established by machine learning methods have been able to realize the calculation of some structural response characteristics and the prediction of riser design parameters quickly, efficiently, and with high accuracy [154,155]. Currently, in the field of unbonded flexible risers, researchers have used data-driven kriging-based methods to predict the collapse pressure of spiral self-locking skeleton layer structures [156]. In addition, a Bayesian inversion framework using phase field modes for type estimation of material parameters during ductile fracture may also have future applications in the study of unbonded flexible risers for composites. The use of machine learning methods to solve complex engineering problems related to riser structures is a popular research direction of common concern at home and abroad at present. However, the possibility of using machine learning and deep learning methods to help solve marine engineering problems is still being continuously explored, and the study of the mechanical properties and failure characteristics of the cross-section of unbonded flexible risers based on the machine learning method will help the design of the future structure of unbonded flexible risers.

## 4. New Types of Unbonded Flexible Risers and Research Hotspots

The development of unbonded flexible risers in recent years has focused more on the functionality of the structure and the need for expansion into the deep sea. Three newer types of unbonded flexible risers are briefly described below.

### 4.1. Integrated Production Bundle, IPB

**Figure 22:**
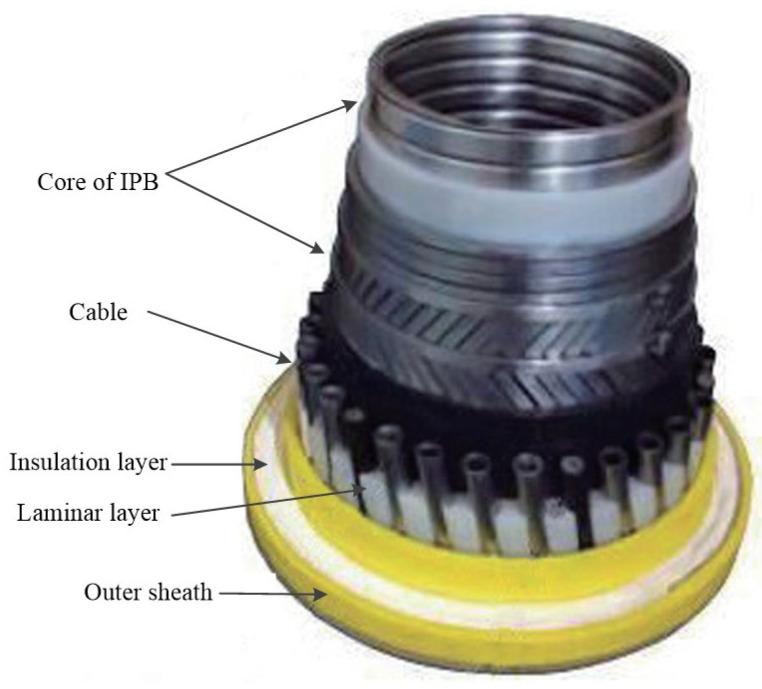
Sketch of an IPB [14].

Technip has developed the integrated production bundle (IPB, as shown in Figure 22) as a deep-sea dynamic riser. The IPB is a combination of a typical riser and an umbilical riser, where the primary role of the IPB is to transfer chemicals or electricity. The IPB combines efficient heating and temperature monitoring for safer and more flexible operation. At the same time, the IPB, for the first time, puts gas lift and electric heating in the same riser, with cables and isolators assembled in the form of S and Z around the core of a standard riser with an inner diameter of 50.8 to 304.8 mm.

Technip installed an IPB dynamic riser with a total length of 13,200 m and an internal diameter of 273.05 mm, electrically heated and gas-lifted, for a total of 1350 m offshore Angola. They also installed 2400 m of IPB dynamic risers with an internal diameter of 254 mm, electrically heated and airlifted, in the same sea area. Meanwhile, a 110 km long riser, with an internal diameter of 152.4 mm and heated IPB, was installed at the Papa-Terra project in Brazil. The project uses innovative distributed temperature sensors to monitor and control the heating of the riser, which heats up the thick oil as it is being transported, reducing the viscosity of the oil and increasing the flow rate and oil production rate.

### 4.2. An Unbonded Flexible Riser with an Anti-H_2_S Layer

When transporting oil or gas with H_2_S, it can diffuse through the polymer liner, and the diffusion mechanism is affected by temperature, H_2_S content, the thickness of the H_2_S-resistant layer, and the permeability of the material. Affected by H_2_S diffusion, the selection of the pressure armor layer and tensile armor layer should consider the compatibility of alloy steel, so it is advisable to choose the steel with high anti-H_2_S performance, but possibly at the expense of its mechanical properties. To achieve the performance requirements of the unbonded flexible riser, it is necessary to increase the wall thickness of steel materials to match the mechanical load, axial pressure, and water depth pressure of the riser. The inner sheath layer is the sealing layer for the transported fluid, and over time, water, CO_2_, and H_2_S will permeate through the inner sheath layer. An H_2_S-resistant layer between the polymer liner layer and the pressure armor layer effectively blocks the diffusion of H_2_S [23].

PEZnO is a thermoplastic polyethylene (PE) matrix composite containing a blend of ZnO and Fe_2_O_3_. ZnO would chemically react with H_2_S penetrating through the inner sheath layer, greatly reducing the penetration rate of H_2_S, thus improving the H_2_S resistance of unbonded flexible risers. Fe_2_O_3_ is the initial purple material and would act as a visual tracer for the reaction with H_2_S. The main reaction of the anti-H_2_S process is as follows:

ZnO + H_2_S → ZnS + H_2_O. (1)

The chemical reaction occurs within a very thin area, known as the reaction zone, and is irreversible. The mechanism of H_2_S resistance in an unbonded flexible riser that incorporates an H_2_S-resistant layer is as follows: the reaction initiates on the inner surface of the H_2_S-resistant layer. Over time, as H_2_S penetrates and diffuses, the thin reaction zone progressively advances. ZnO on the inner surface of the H_2_S-resistant layer reacts with H_2_S to form ZnS, marking the formation of the PEZnS region. In areas where H_2_S has not yet penetrated, the ZnO remains unreacted, preserving the integrity of the H_2_S-resistant layer [12].

The advantages of using an H_2_S-resistant layer are as follows: (1) mass reduction: the unbonded flexible riser has 30% less mass with the same structural dimensions, and the H_2_S-resistant steel can be replaced with non-H_2_S-resistant steel, which improves the anti-H_2_S performance of the riser, reduces the cross-sectional area of the riser, and reduces the tensile load on the top, which is even more advantageous in ultra-deep water applications; (2) extended service life: the H_2_S-resistant riser can transport H_2_S-containing liquids with a partial pressure of 0.1 MPa for 20 years.

The average temperature of the H_2_S-resistant layer in conventional static risers is 70 °C. The focus of current research is to extend the application temperature of the H_2_S-resistant layer (100 °C, 130 °C); thus, it can be adapted to dynamic high-temperature environments. Direct and indirect monitoring techniques for evaluating the consumption of H_2_S-resistant materials are also a focus of the research. The large amount of technical information obtained by actually monitoring and collecting the results of the use of the H_2_S-resistant layer allows for a more precise design of the H_2_S-resistant layer, thus extending the service life of the riser.

### 4.3. Unbonded Flexible Risers with Composite Armor Layers

Composites usually consist of matrix material and reinforcing material (usually fibers) and have been used for many years in the field of offshore engineering. They are usually structured in the form of a regular cylindrical shell structure and are commonly used in structures such as reinforced thermoplastic pipes and marine hoses [157,158]. The structural properties of composites are more complex than those of isotropic materials and are usually simplified to anisotropic materials for treatment and analysis.

With the development of the operating water depth of the offshore oil and gas industry to ultra-deep water, the suspended weight and fatigue performance of risers have gradually become the dominant factors in their design [24,25]. Additionally, when the operating water depth exceeds 2000 m, the supporting equipment, such as pipe-laying vessels and buoyant counterweights, would be difficult to satisfy the laying demands of the traditional steel pipelines of the traditional unbonded flexible risers. The use of composites with less mass can be a good solution to this challenge.

Composites cannot only reduce the overall mass of unbonded flexible risers [26,27,28] but they have good fatigue and corrosion resistance as well and can reduce the cost by approximately 15% compared to conventional steel tensile armor layers under the same conditions. Riser manufacturers such as Technip have carried out real-scale dynamic tests in ultra-deep waters in West Africa, as well as in Brazil and the Gulf of Mexico, to verify the feasibility of containing composite tensile armor layers.

The excellent fatigue resistance of carbon fiber composites under external loading makes them very suitable for deep-water dynamic risers. In acidic environments containing H_2_S, the structural layers of normal flexible risers have to be made of hydrogen sulphone-resistant steel with lower mechanical properties. In typical acidic service conditions and ultra-deep-water conditions, flexible risers with a steel tensile armor layer with an inner diameter of 228.6 mm require a mass of 265 kg per meter and a top tension of 3110 kN. The advantages of using carbon fiber as the tensile armor layer include the following: higher strength versus the mass ratio of the carbon fibre tensile armor layer, which allows a significant reduction of the riser mass under the same structural performance conditions, up to 139 kg per meter of riser; improving the fatigue performance of the riser, in particular, high strength versus the mass ratio reduces the buoyant counterweight on the top riser, thus, only a small vessel is required for its installation and transportation, which can reduce the costs of installing the riser system by reducing the size of the vessel. The latter advantage that is even more evident in the installation of riser systems in ultra-deep water. Meanwhile, the carbon fiber tensile armor layer is insensitive to H_2_S, which can reduce the corrosion of the riser by oilfield chemicals and seawater.

Selection of the optimum fibers, resins, and manufacturing processes is the key to creating carbon fiber tensile armor layers. Superior carbon fiber materials can have tensile strengths in excess of 3000 MPa. The strength of carbon fiber tensile armor layers is more than twice as strong as that of high-strength steel tensile armor layers, but only 1/5 the mass. The corresponding comparison is presented in Table 3.

The most significant advantage of carbon fiber tensile armor layers is the reduction in tensile loads at the top of the riser, which offers the possibility of reducing the buoyancy module, making it cost-effective for applications in ultra-deep water.

The material properties of composites are more complex than those of isotropic homogeneous materials, and the mechanical properties of the tensile armor layer of composites, taking into account the complex spatial geometry of the tensile armor layer, are even more complex. As a result, few corresponding studies have been carried out so far. Based on the theoretical model of Kebadze [60], Liu et al. [24,25] extended the theoretical model of the traditional homogeneous tensile armor layer to a theoretical model that can simultaneously take into account the tensile armor layer of composites and the steel tensile armor layer and carried out the calculation of the mechanical properties of the composite tensile armor layer in different working conditions by establishing a numerical model with detailed geometric properties. Consequently, the accuracy of the theoretical and numerical methods was mutually verified.

## 5. Research Prospect

Unbonded flexible risers are structurally complex. In recent years, industrial and academic research interest in unbonded flexible risers has increased, and with the development of the offshore oil and gas industry, some new technologies and materials have been gradually introduced into the design and manufacture of unbonded flexible risers. Expanding the application range of unbonded flexible risers is accompanied by some new problems that need to be solved. Combining the limitations of this paper and the future development trend of unbonded flexible risers, it is believed that in-depth research can be carried out on the following aspects in the future:

1. Experimental research on the cross-sectional mechanical properties of unbonded flexible risers with composite tensile armor layers: Carrying out model tests of unbonded flexible risers with composite tensile armor layers is of great significance for understanding the structural characteristics of composite tensile armor layers and carrying out the design of unbonded flexible risers. In addition, the influence of axisymmetric loads, such as internal and external pressures, on the bending hysteresis effect of unbonded flexible risers is inconclusive; thus, carrying out research on the bending characteristics of unbonded flexible risers with the action of internal and external pressure loads can lead to a better understanding and mastery of the hysteresis characteristics of unbonded flexible risers.
2. Failure characteristics of composite tensile armor layers: The failure characteristics of composites and isotropic materials are different. Due to their special structural form, the destructive stress in the axial and radial directions of composites differ greatly. While focusing on the axial tensile destruction of the tensile armor layer of the composite material, it is also necessary to be alert to their destruction under the action of internal and external pressure loads. At the same time, the tensile armor layer might undergo complex radial and lateral failure under axial compression. The replacement of the original steel tensile armor layer with a composite tensile armor layer might also have a certain impact on the failure characteristics. It is recommended that the failure characteristics of composite tensile armor layers under axial force and internal and external pressure loads should be studied by experimental methods and simulated by reasonable numerical methods.
3. Theoretical modeling of radial and lateral failure of tensile armor layers: The failure mode of the tensile armor layer structure under axial compression is relatively complex. The current theoretical model is based on the straight and curved beam theory to solve the equilibrium equation of the helical tendon under axial compression. The present theoretical method has made many assumptions, and the relative deviation from the test and numerical calculation results is relatively large. It is suggested to consider the failure path of the helical tendon reasonably under axial compression, thus predicting the ultimate bearing capacity of the tensile armor layer under axial compression more accurately.
4. Dynamic response of unbonded flexible risers: Due to the nonlinear bending characteristics of unbonded flexible risers, their dynamic response analysis is very complex and is mostly calculated using Orcaflex commercial software, version 11.4 (OrcaFlex—dynamic analysis software for offshore marine systems (orcina.com)). However, the treatment of unbonded flexible risers using Orcaflex software (version 11.4) is relatively simple and cannot take into account the elongation conditions of risers, which might have a great impact on the results of the dynamic calculations under high tension. It is proposed to carry out the dynamic response study of unbonded flexible risers based on the slender theory, establishing the force balance equation of unbonded flexible risers and the control equation considering the elongation condition. Meanwhile, the bending nonlinearity is taken into account, and the bending stiffness matrix of the unbonded flexible riser element is updated in real-time to carry out the analysis of the overall dynamic response characteristics of unbonded flexible risers in the marine environment.
5. Effect of temperature on the mechanical properties of unbonded flexible riser sections: The material properties of the layer structure in unbonded flexible risers, especially the polymer structure, are strongly influenced by temperature, and the effect of temperature on the material should be taken into account to affect the cross-section mechanical properties of unbonded flexible risers.
6. Application of artificial intelligence and machine learning: The rise of artificial intelligence (AI) and machine learning (ML) opens up a range of new avenues for solving engineering problems, with rising applications in the field of offshore industry. AI and ML would aid in the structural design of future unbonded flexible risers.

## 6. Conclusions

This paper presents the development of unbonded flexible risers on new material, types of layers, and mechanical properties. The following conclusions have been drawn:

1. The theoretical models of unbonded flexible risers are all established based on a large number of strict assumptions. Considering the geometric relationship and based on the functional principle, the load–strain relationship of unbonded flexible risers under axisymmetric loading is basically linear. The theoretical model of unbonded flexible risers under the bending moment is more complicated compared to that under axisymmetric load; the helical tendons of the tensile armor layers are free to slide after overcoming interlayer friction, resulting in a decrease in bending stiffness.
2. The establishment of a three-dimensional numerical model of an unbonded flexible riser under axisymmetric loading has roughly gone through development, simplifying part of the layer structure and taking into account the detailed geometrical properties of each layer. Additionally, due to the existence of a large number of geometrical nonlinearities and contact nonlinearities within the numerical model, it is generally solved by explicit algorithms. Applying axisymmetric loads to the numerical model of the unbonded flexible riser is required before applying the bending moment to determine the initial prestressing effect between adjacent layers.
3. The introduction of composite materials into the manufacturing of unbonded flexible risers is the current development trend of marine unbonded flexible risers. The mechanical properties of unbonded flexible risers containing composites are extremely complex, although some riser manufacturers have given verification tests of unbonded flexible risers containing composite tensile armor layers. However, the relevant research on the mechanical properties of the cross-section of the composite tensile armor layer is still lacking, and carrying out the relevant research is conducive to the breakthrough of the key technologies for the development of marine oil and gas resources.
4. Unbonded flexible risers technology is poised to become even more integral to the marine industry. As deepwater exploration continues to expand, the demand for high-performance, unbonded flexible risers will grow. The long-term vision includes the development of unbonded flexible risers that are not only more robust and reliable but also adaptable to various marine environments and operational demands.

In conclusion, the development of unbonded flexible risers is a dynamic field with ongoing advancements in materials science and computational modeling. The insights gained from this study provide a solid foundation for future research and development efforts, ultimately contributing to the advancement of marine oil and gas extraction technologies.

## Figures and Tables

**Table 1:** Geometry, specification parameters, and scope of the application of a carcass layer [13].

Cross-Sectional Area/mm^2^	Thickness/mm	Scope of Application (Riser I.D. Size)/mm
0.62 × 28	3.25	50.8~76.2
1.00 × 36	5.00	76.2~127.0
1.20 × 40	6.00	152.4~203.2
1.40 × 55	7.00	203.2~304.8
1.60 × 55	8.00	203.2~304.8
2.00 × 72	10.00	304.8~421.6

**Table 2:** Specification parameters and scope of the application of a ‘Z’-type pressure armor layer [13].

Cross-Sectional Area/mm^2^	Scope of Application (Riser I.D. Size)/mm
10.6×4.8	50.8~76.2
14.4×6.4	76.2~127.0
18.0×8.0	152.4~203.2
22.4×10.0	203.2~304.8
27.0×12.0	203.2~304.8

**Table 3:** Comparison of different tensile armor layers [16].

Material	Tensile Strength/MPa	Elongation/%	Young’s Modulus/GPa	Densities/g·cm^−3^
High strength steel	3.25	≥5	210	7.8
Corrosion-resistant steel	5.00	≥10	210	7.8
Composite	10.00	≥1.8	160	1.7

## Data Availability

Data are contained within the article.
